# Socioeconomic Status Impact on Diet Quality and Body Mass Index in Eight Latin American Countries: ELANS Study Results

**DOI:** 10.3390/nu13072404

**Published:** 2021-07-14

**Authors:** Georgina Gómez, Irina Kovalskys, Ana Carolina B. Leme, Dayana Quesada, Attilio Rigotti, Lilia Yadira Cortés Sanabria, Martha Cecilia Yépez García, María Reyna Liria-Domínguez, Marianella Herrera-Cuenca, Regina Mara Fisberg, Agatha Nogueira Previdelli, Viviana Guajardo, Gerson Ferrari, Mauro Fisberg, Juan Carlos Brenes, on behalf of the ELANS Study Group

**Affiliations:** 1 Departamento de Bioquímica, Escuela de Medicina, Universidad de Costa Rica, San José 11501-2060, Costa Rica; dayana.quesada@ucr.ac.cr; 2 Carrera de Nutrición, Facultad de Ciencias Médicas, Pontificia Universidad Católica Argentina, Buenos Aires C1107AAZ, Argentina; ikovalskys@gmail.com; 3 Family Relations and Applied Nutrition, University of Guelph, Guelph, ON N1G 2W1, Canada; acarol.leme@gmail.com; 4 Departamento de Nutrição, Faculade de Saúde Publica, Universitade de São Paulo, São Paulo 01246-904, Brazil; rfisberg@usp.br (R.M.F.); agatha.usp@gmail.com (A.N.P.); 5 Centro de Excelencia em Nutrição e Dificuldades Alimentaes (CENDA), Instituto Pensi, Fundação José Luiz Egydio Setubal, Hospital Infantil Sabará, São Paulo 01228-200, Brazil; mauro.fisberg@gmail.com; 6 Centro de Nutrición Molecular y Enfermedades Crónicas, Departamento de Nutrición, Diabetes y Metabolismo, Escuela de Medicina, Pontificia Universidad Católica, Santiago 8330024, Chile; arigotti@med.puc.cl; 7 Departamento de Nutrición y Bioquímica, Pontificia Universidad Javeriana, Bogotá 110231, Colombia; ycortes@javeriana.edu.co; 8 Colegio de Ciencias de la Salud, Universidad San Francisco de Quito, Quito 17-1200-841, Ecuador; myepez@usfq.edu.ec; 9 Área de Investigación, Instituto de Investigación Nutricional, La Molina, Lima 15026, Peru; rliria@iin.sld.pe; 10 Facultad de Ciencias de la Salud, Universidad Peruana de Ciencias Aplicadas, Lima 15023, Peru; 11 Fundación Bengoa, Caracas 1070, Venezuela; manyma@gmail.com; 12 Centro de Estudios del Desarrollo, Universidad Central de Venezuela (CENDES-UCV), Caracas 1010, Venezuela; 13 Instituto Para la Cooperación Científica en Ambiente y Salud (ICCAS), Buenos Aires C1059ABF, Argentina; vguajardo@iccas.org.ar; 14 Escuela de Ciencias de la Actividad Física, el Deporte y la Salud, Universidad de Santiago de Chile (USACH), Santiago 7500618, Chile; gerson.demoraes@usach.cl; 15 Departamento de Pediatria, Universidade Federal de São Paulo, São Paulo 04023-061, Brazil; 16 Instituto de Investigaciones Psicológicas, Centro de Investigación en Neurociencias, Universidad de Costa Rica, San José 10501-2060, Costa Rica; juan.brenessaenz@ucr.ac.cr

**Keywords:** diet quality, micronutrients, socioeconomic status, Latin America, nutrition surveys

## Abstract

Poor health and diet quality are associated with living within a low socioeconomic status (SES). This study aimed to investigate the impact of SES on diet quality and body mass index in Latin America. Data from the “Latin American Health and Nutrition Study (ELANS)”, a multi-country, population-based study of 9218 participants, were used. Dietary intake was collected through two 24 h recalls from participants of Argentina, Brazil, Chile, Colombia, Costa Rica, Ecuador, Peru and Venezuela. Diet quality was assessed using the dietary quality score (DQS), the dietary diversity score (DDS) and the nutrients adequacy ratio (NAR). Chi-squared and multivariate-variance analyses were used to estimate possible associations. We found that participants from the low SES consumed less fruits, vegetables, whole grains, fiber and fish and seafood and more legumes than those in the high SES. Also, the diet quality level, assessed by DQS, DDS and NAR mean, increased with SES. Women in the low SES also showed a larger prevalence of abdominal obesity and excess weight than those in the middle and high SES. Health policies and behavioral-change strategies should be addressed to reduce the impact of socioeconomic factors on diet quality and body weight, with gender as an additional level of vulnerability.

## 1. Introduction

The Latin American region is comprised of twenty countries, home to 642 million people [1]. Evidence has shown that Latin American countries are experiencing a nutrition transition, moving from underweight to overweight/obesity [2]. Globally, 52% of adults are overweight or obese [3]. A representative study with 9218 individuals living in urban areas of eight LA countries showed that 59.6% were overweight or obese, with Chile showing the highest (68.6%) and Colombia the lowest (50.7%) prevalence [4]. Obesity is driven by key behavioral and lifestyle risk factors that are in turn modified by socio-demographic and epidemiological changes that have occurred in LA populations over the past decades [5,6].

Several epidemiological studies have shown that differences in socio-economic and demographic characteristics carry a considerable impact on lifestyle risk factors [7]. For instance, individuals from low socio-economic status tend to have unhealthier lifestyle, such as unhealthy diets and decreased time spent on physical activity [8]. Moreover, differences within individual socio-economic status (SES) exist. Concurrently, studies conducted across different low- and middle-income countries suggested important socio-economic disadvantages, as contextual factor, with independent effects on dietary factors [9,10]. More research is needed to understand socio-economic status from a perspective of comparing different studies from low- and middle-income countries to better understand the mechanism underlying socio-economically patterned diets. 

Poor health and diet quality are associated with individuals pertaining to a low socio-economic status (SES), as compared to those from middle or high SES [7,8]. Other socio-demographic characteristics can be associated with diet quality, such as the place of birth, education background, race/ethnicity and family aspects (such as the number of kids and their age) [8]. Evidence from LA countries has shown an increased intake of discretionary food items, such as those rich in saturated fats, added sugars and sodium and inadequate intakes, as compared with recommended levels of fruit and vegetables, milk and dairy products and whole grains [9]. Consequently, individuals living in LA countries might experience a shortfall of micronutrients [10]. 

Since the focus is on foods and diet quality rather than the effect of single nutrients on health, understanding how diet quality is associated with socio-demographics and micronutrients intake has become relevant to nutrition research [11,12,13]. However, there is a gap in research that has been undertaken in low- and middle-income countries, including LA countries. Multi-country surveys provide important information on health behaviors of a population and sub-groups. Information including inadequacy of dietary intake in certain population groups is needed to identify relevant fields of action for public health policies and behavioral-change interventions [13]. The Latin American Health and Nutrition Study (acronym in Spanish and Portuguese, ELANS) [14] provides an opportunity to understand these gaps. The available information on diet quality of LA individuals older than 15 years old may help identify possible associations with socio-demographic characteristics that can impair on inadequate intakes of micronutrients. The aim of this study was to understand the impact of SES on diet quality and body mass index in a representative sample of Latin American individuals older than 15 years old.

## 2. Materials and Methods

### 2.1. ELANS Overview

The ELANS is a multi-country, household, cross-sectional study that uses a multistage stratified by geographical location (only urban cities), sex, age and SES of non-institutionalized individuals of eight LA countries. Urban areas were included, rather than rural areas, to provide a population homogeneity and because most of the included countries have up to 90% of individuals living in these areas. An overview of the ELANS study including the purpose, plan and operations, sample design, weighting procedures, analytic guidelines and response rates and population totals is available elsewhere [14]. In addition, a protocol study on the standardization for dietary intake analysis for the ELANS study has been published [15]. The survey examined approximately 9000 persons from September 2014 to June 2015. Trained interviewers collected data via self-reported questionnaires (e.g., dietary and physical activity recalls) and objective measurements (e.g., weight and height) according to standardized procedures. The ELANS was approved by the Western Institutional Review Board (#20140605) and registered in clinicaltrials.gov (*n*° NCT02226627). Local research institutes ethics review boards from each country also needed to approve the study. All participants provided written informed consent/assent form to participate in this survey.

### 2.2. Study Sample

Data from adolescents and adults aged 15–65 years old participating in the ELANS were analyzed, resulting in a final analytic sample of 9218 after excluding those with unreliable records as defined by ELANS staff. The unweighted responses rates for the participants from each country were: 13.73% Argentina, 21.70% Brazil, 9.54% Chile, 13.34% Colombia, 8.66% Costa Rica and Ecuador, 12.07% Peru and 12.28% Venezuela. The sample was randomly selected of primary and secondary sampling units. The households were selected within each secondary sampling unit, via systematic randomization. The choice of the participant within a household was conducted with 50% of the sample next birthday and 50% last birthday methods, controlling for sex, age and SES. The representative sample size was established with a confidence level of 95% and a maximum error of 3.49%. Sample weighting was applied for each country accounting for key variables of interest: sex, age and SES. The ELANS employs protocols and procedures that ensure confidentiality and protect individual participants from identification.

### 2.3. Sociodemographic Characteristics

Independent variables included sex (male vs. female), age group and SES. Participants were grouped into four age categories: adolescents (15–19 years), according to the World Health Organization [16], and adults (20–34 years, 35–49 years and 50–65 years). SES was evaluated by questionnaire, using a country-dependent format, based on the governmental reference of each country and according to national population census and on the legislative requirements, or established local standard layouts. These questionaries included at least the following items: head of house’s income, occupation and education, family members or number of persons living in the house, housing and accommodation status (house ownership, house area, type of home, construction materials, number of rooms in house, water, gas and electricity supply) and home appliances and personal assets, such as vehicles, computers and other devices ownership. Further details on weighting and scoring of items in each country have been previously published [17,18,19,20,21,22,23,24] Afterward, three levels of classification were stablished, according to the equivalent characteristics of all countries, into low, middle and high.

### 2.4. Dietary Assessment

Dietary intake data were obtained from two non-consecutive in-person 24 h dietary recall (24hR) using an automated multiple-pass method [25], including both weekdays and weekend days, with a proportional distribution of days among the sample, in order to capture the day-to-day variation in food consumption. A photographic album of common foods of each country and household utensils were used to estimate portion sizes. Energy and nutrients from the foods and beverages were calculated with the help of the software Nutrition Data System for Research version 2013 (NDS-R, University of Minnesota, Minneapolis, MN, USA). Local foods reported by the participants were previously standardized matching the equivalence of energy and nutrients from the NDS-R database using local food composition tables and nutrition facts panels. A concordance rate of at least 80–120% for energy and nutrients values was needed to establish an equivalence between local and foods available in the NDS-R database. Energy and nutrients were estimated for usual dietary intakes using the Multiple Source Method (MSM) (https://msm.dife.de/ (accessed on 16 July 2015)) [26], which converts individual intakes derived from the two 24hR to usual distributions. Trained interviewers collected the recall data in Portuguese (in the case of Brazil) and in Spanish (for the other Latin American countries). Participants were asked to report all the foods and beverages eaten on the previous day.

### 2.5. Diet Quality

Several indexes for assessing diet quality have been proposed for both adolescents and adults [27], such as the Healthy Eating Index (HEI), Alternate Healthy Eating Index (AHEI) and Dietary Approaches to Stop Hypertension Score (DASHS). However, the dietary quality score (DQS), proposed by Imamura et al. [28] seems to be the most appropriate approach for the ELANS context. Briefly, the DQS was based on dietary information derived from individual-based national surveys as part of the Global Burden of Disease Nutrition and Chronic Diseases Expert Group (NutriCoDE), representing 88.7% of the global adult population. This approach evaluates the consumption of key dietary items, adjusted for 2000 kcal per day, modelling two different dietary patterns: (i) healthy items and (ii) unhealthy items. Healthy items were measured by relatively high consumption of 10 food groups, i.e., fruits, vegetables, beans and legumes, nuts and seeds, whole grains, milk, polyunsaturated fats (PUFAs), fish, plant omega 3 and dietary fiber. Unhealthy items were measured for relatively low consumption of seven groups, i.e., unprocessed read meats, processed meats, sugar sweetened beverages (SSB), saturated fat, trans fat, dietary cholesterol and sodium. Finally, a third pattern incorporates both healthy and unhealthy items and creates a sum for a total score. 

To obtain scores for each pattern, the mean age-, sex-, country-specific intakes of each dietary factor were divided into quintiles. Each quintile was assigned an ordinal score. Higher scores were given to quintiles with higher mean intakes of healthy foods (from 1 to 5). For unhealthy foods, higher scores were given to quintiles with lower mean intakes (from 5 to 1 points). For each population stratum, scores across different dietary items were summed to obtain the total score for each of three dietary pattens: healthy items, unhealthy items and all items combined. Scores were based on the intake of 17 items and highest scores for the healthy and unhealthy patterns were 50 and 35 points, respectively. All items were summarized to obtain the overall DQS of 85 points. All DQS were standardized to a 100-point scale, the higher scores meaning healthier diets. A detailed analysis on DQS on the ELANS study population has been previously published [29].

### 2.6. Dietary Diversity

For the dietary diversity score (DDS), all food items reported to have been consumed during the first 24 h recall were classified into ten food groups, according to the Minimum Dietary Diversity Score for Women (MDD-W), proposed by FAO [30]: (1) starchy staples (grains, with roots and tubers and plantains); (2) meat, poultry and fish; (3) dark green leafy vegetables; (4) other vitamin A-rich fruits and vegetables; (5) other vegetables; (6) other fruits; (7) pulses (beans, peas and lentils); (8) dairy; (9) eggs; (10) nuts and seeds. If the consumption of each food group was at least 15 g/day, 1 point (if consumed) was assigned, or 0 points (if intake of that specific food group was less than 15 g/day) were assigned, for a maximum score of 10 points.

### 2.7. Nutrient Adequacy

The prevalence of adequacy, for micronutrients, was determined using the Estimated Average Requirements (EAR), from the Institute of Medicine. They were used because they are a recommended standard parameter to estimate the prevalence of inadequate nutrient intake within a group [31]. The estimation of 17 out 18 micronutrients was calculated based on the nutrient adequacy ratio (NAR) for vitamins A, C, D and E, calcium, iron, thiamin, riboflavin, niacin, cobalamin, pyridoxine, zinc, magnesium, copper, folate, phosphorus and selenium. The only exception was sodium, as there is insufficient evidence of casual relationship between sodium and indicator of adequacy, as well as evidence of an intake–response relationship for this nutrient to establish an EAR [32]. The NAR value for a given nutrient is the ratio of a participant’s current nutrient intake to the EAR for the corresponding sex and age category [33]. Values close to 1 for the NAR that recommended nutrient intake were achieved or exceeded. Considering that a nutrient with a high NAR cannot be compensated by a nutrient with a low NAR, all NARs were truncated at 1. The mean adequacy ratio (MAR) was calculated as the sum of all NARs divided by the number of nutrients assessed. The cut-off point of 0.6 was used as adequacy ratio for nutrient adequacy to ensure comparability with previous multi-country analyses [7,23]. MARs were compared by age group, country, SES, weight status and dietary quality scores accomplishment. DDS has been previously validated as a proxy of micronutrients adequacy in women from the ELANS study sample [34].

### 2.8. Weight Status

Weight and height were carried out by trained research assistants following a standardized protocol for anthropometric procedures and collection drawn up by the ELANS group [14]. Participants were asked to wear normal, light indoor clothing and remove their shoes and other personal belongings. Body weight was measured in kilograms, to the nearest 0.1 kg with portable digital scales. Height was measured in centimeters with stadiometers and the reading was taken to the last completed 0.1 cm. Body mass index (BMI) was calculated and weight status was defined according to participants age group. Adolescents’ weight status was classified according to WHO z-scores for age and sex [35]. For those over 18 years old, BMI was defined following the WHO BMI classification: underweight, if BMI was ≤18.5 kg/m^2^, normal weight, if BMI > 18.5–24.99 kg/m^2^, overweight, if BMI ≥ 25.0–29.9 kg/m^2^, and obese, if BMI ≥ 30.0 kg/m^2^ [36]. Waist circumference (WC) cut-off was established at ≥102 cm for men and ≥88 cm for women [37]. For a further analysis, we classified participants with no excess weight (underweight and normal weight) and with a waist circumference below the cut-off point as lean with normal weight and those overweight and obese, with waist circumference above the cut-off point, as abdominal obesity and excess weight.

### 2.9. Data Analysis 

Data were analyzed using the Statistical Package for Social Sciences (SPSS) software program (version 23, SPSS Inc., Chicago, IL, USA). Data were reported as mean ± standard deviations (SD). The between-group comparisons (i.e., socioeconomic levels as an independent variable) of dependent variables (e.g., food groups, micronutrients, diet quality indicators and BMI) were analyzed with factorial multivariance analyses (MANOVA), followed by Tukey’s Highest Statistical Difference (HSD) post hoc test, when appropriate. The MANOVA analyses were controlled by country, sex and age according to the dependent variables analyzed. For instance, as diet quality indicators were compared among SESs within each country, only sex and age were used as control covariables. In the case of the BMI, we included sex as a cofactor, in addition to the SES factor, as sex is the most important variable determining the differences in body composition and BMI. Thus, we used country and age as covariables in that analysis. Partial eta squared coefficients (η^2^_p_) were estimated as an index of the effect size. The Kolmogorov–Smirnov test was used to evaluate the normality assumption of the distribution. Those variables that were not normally distributed followed two further steps. First, we performed non-parametric tests (e.g., Kruskal–Wallis tests) to check whether the violation of the assumption yielded distinct significance levels compared to those obtained with the parametric test. Second, we transformed the variables using the square-root transformation, which is appropriate for data containing zero values. Then, we repeated the parametric tests and compared the *p* values with those obtained with the untransformed data. If there were no major differences in the significance level among the three analyses, we kept the parametric data, because they allowed us to run multivariate analysis controlled by many variables of interest and also provided the eta square coefficients. A chi-square test (χ_2_) was used to estimate the significant differences in the distribution of participants among sex, countries, age in intervals and nutritional status (i.e., persons with a waist circumference below cut-off point and normal weight vs. those with abdominal obesity and excess weight). In all analyses, *p* < 0.05 values were considered statistically significant.

## 3. Results

### 3.1. Distribution of Sociodemographic Variables among the Socioeconomic Levels

The distribution of the total ELANS sample, according to socioeconomic status (SES), differed significantly (χ^2^_(14,9218)_ = 753.79, *p* = 0.0001). Table 1 shows how many individuals in each country pertained to different levels of income status, as compared to the entire ELANS sample. Countries were ranked according to their percentage of individuals in the low SES. Therefore, Brazil, Venezuela and Colombia showed the highest percentage of low SES (ranging from 19.1 to 16.2%). In contrast, Chile, Ecuador and Costa Rica showed the lowest percentage of low SES (ranging from 8.6 to 5.5%). There were also significant differences between sex and SES (χ^2^_(2,9218)_ = 9.27, *p* = 0.01). A detailed analysis showed that such differences were observed only in the low SES, in which there were significantly more women than men (χ^2^_(1,47968)_ = 26.12, *p* = 0.0001). No significant differences among age intervals were observed.

### 3.2. Consumption of Food Groups among the Socioeconomic Levels

The consumption of the food groups is shown in Table 2. The MANOVA analyses comparing the SESs were controlled by country, sex and age, as those factors have their own impact on food intake. The comparison among SESs yielded statistical differences for all groups, with no differences in the energy intake. According to the effect size (i.e., percentage of variance explained by the SES), the food groups were ranked (Table 2). The food groups that varied significantly among the three SESs were vegetables (*F*_(2,9212)_ = 86.406, *p* = 0.0001), fruits (*F*_(2,9212)_ = 61.172, *p* = 0.0001), whole grains (*F*_(2,9212)_ = 30.5646, *p* = 0.0001) and fiber (*F*_(2,9212)_= 25.810, *p* = 0.0001). For all these groups, consumption increased with SES (HSD, all *p* values < 0.05). Red meat consumption also differed among the three SESs (*F*_(2,9212)_ = 14.907, *p* = 0.0001), with the lowest consumption being observed in the high SES, followed by the low and the middle SES (HSD, all *p* values < 0.05). The consumption of processed meat (*F*_(2,9212)_= 5.253, *p* = 0.005) was also lower in high SES, with the other SESs showing similar values between each other. Fish and seafood (*F*_(2,9212)_ = 9.767, *p* = 0.0001) groups were more consumed in the high SES, relative to the other two levels, which did not differ between one another (HSD, all *p* values < 0.05). Next, it was the consumption of dairy (*F*_(2,9212)_ = 14.284, *p* = 0.0001) and nuts and seeds (*F*_(2,9212)_ = 3.017, *p* = 0.049) groups, which were lower in the low SES, (HSD, all *p* values < 0.05), with no other differences detected between the middle and high SES. Finally, there were the legumes (*F*_(2,9212)_ = 16.615, *p* = 0.0001) and the SSB (*F*_(2,9212)_ = 6.4203, *p* = 0.002) groups, which showed a particular pattern of consumption with the middle SES showing the highest and the lowest consumption, respectively. There were no significant differences between low and high SESs.

### 3.3. Micronutrient Adequacy Ratio (NAR) among the Socioeconomic Statuses

The NAR values were compared among SESs by means of MANOVA analyses controlled by country, sex and age, as those factors have their own impact on micronutrients intake (Table 3). Vitamins C (*F*_(2,9212)_ = 45.436, *p* = 0.0001), A (*F*_(2,9212)_ = 43.019, *p* = 0.0001) and D (*F*_(2,9212)_ = 32.192, *p* = 0.0001) differed significantly among all SESs (HSD, all *p* values < 0.05), with the levels of the vitamins being proportional to the SES. In the case of magnesium (*F*_(2,9212)_ = 14.513, *p* = 0.0001, η^2^_p_ = 0.003) and pyridoxin (*F*_(2,9212)_ = 5.465, *p* = 0.0003, η^2^_p_ = 0.001), their levels were significantly higher in the high SES, relative to the other two SES (HSD, all *p* values < 0.05), which did not differ between one another. In contrast, calcium (*F*_(2,9212)_ = 11.573, *p* = 0.0001, η^2^_p_ = 0.003), copper (*F*_(2,9212)_ = 5.925, *p* = 0.0001, η^2^_p_ = 0.001), iron (*F*_(2,9212)_ = 5.321, *p* = 0.0005, η^2^_p_ = 0.001) and riboflavin (*F*_(2,9212)_ = 4.020, *p* = 0.018, η^2^_p_ = 0.001) were significantly lower in the low SES, as compared with the other two SESs (HSD, all *p* values < 0.05), which did not vary between each other. Although the cobalamin (*F*_(2,9212)_ = 3.197, *p* = 0.041, η^2^_p_ = 0.001) and thiamin (*F*_(2,9212)_ = 3.898, *p* = 0.02, η^2^_p_ = 0.001) levels were slightly lower in the low SES, relative to the other two SES, the significant differences were only detected between the low vs. middle SESs (HSD, all *p* values < 0.05), perhaps due to the large variability observed within the high SES. Finally, the MAR score (*F*_(2,9212)_ = 45.379, *p* = 0.0001, η^2^_p_ = 0.01) differed significantly among all SESs (HSD, all *p* values < 0.05), with the higher the SES, the higher the MAR. 

### 3.4. Diet Quality Indicators among the Socioeconomic Levels

The DQS, DDS and MAR variables were compared among the SESs following MANOVA analyzes for the whole sample and per country. The means, SD, *p* values and eta squared coefficients are shown in Table 4. There were significant differences for the total ELANS sample, with all the three SESs differing among them (HSD, all *p* values < 0.05), in which the higher the SES, the higher the scores. Argentina was the only country in which the three variables were significantly different, when compared by the SES. DQS also increased significantly with SES in Chile, Ecuador and Venezuela (HSD, all *p* values < 0.05). The same tendency was observed in Costa Rica, Brazil and Colombia, with no significant differences detected. In Peru, conversely, the DQS decreased with SES (HSD, all *p* values < 0.05). Regarding the DDS, we found that Costa Rica, Brazil and Colombia had significantly lower levels in the low SES (HSD, all *p* values < 0.05), whereas in Chile, Ecuador and Venezuela no significant differences were observed. Similar to the DQS, in Peru, the DDS decreased with SES, with no significant differences observed for the DDS. Finally, MAR values increased with SES in all countries (*p* values < 0.05). According to the size effect, the disparity in diet quality indicators among the SES was higher for Argentina, Costa Rica, Brazil, Chile and Colombia, whereas the gap among SESs was the smallest for Ecuador and Venezuela. In terms of the ability of the three variables to discriminate among the SES per country, the best variable was the MAR (differed 8 out of 8), followed by the DDS (differed 4 out of 8) and, finally, the DQS (differed 3 out of 8). 

### 3.5. BMI among the Socioeconomic Status

The BMI was compared among SESs by means of MANOVA analyses, controlled by country, sex and age (Table 5). In the overall sample, the BMI did not differ among the SESs, with BMI values being quite similar, around 27 kg/m^2^. Among the covariables added to the analysis, sex showed to be an important factor in determining BMI differences. A detailed analysis showed that women had significantly higher BMI than men in the middle (6.17%; *F*_(2,9296)_ = 9.762, *p* = 0.0001, η^2^_p_ = 0.002) and low SESs (2.87%, *F*_(2,9296)_ = 9.762, *p* = 0.0001, η^2^_p_ = 0.002), but not in high SES (1.40%; *F*_(2,9296)_ = 1.026, *p* = 0.311, η^2^_p_ = 0.001). As BMI correlated positively with waist circumference (total sample: *r* = 0.837, *p* = 0.0001) in all SESs (range: *r* = 0.836–0.846, all *p*-values < 0.0001), we compared it among SESs, while controlling by country, sex, age and BMI. The estimated means, after substracting the effect of those variables, assumed the following values: low SES = 88.14 ± 3.11; middle SES = 88.39 ± 7.32; high SES 88.43 ± 16.95. There were no significant differences among the SESs.

**Figure 1:**
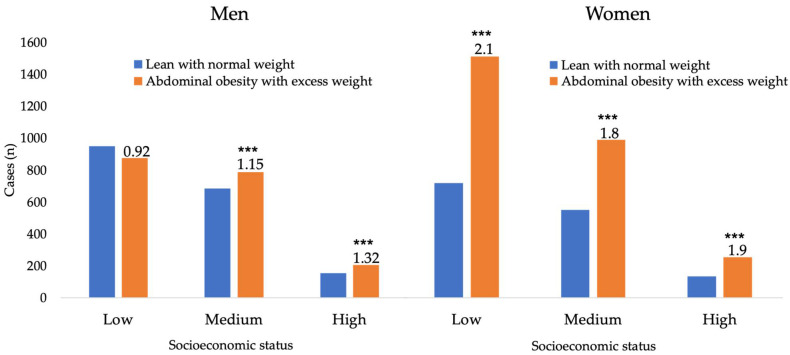
Proportion of men (**left**) and women (**right**) with or without abdominal obesity and excess weight among the socioeconomic statuses. *** *p* < 0.0001: lean body and normal weight vs. abdominal obesity and excess weight. Significant differences correspond to chi-squared analysis within each category of socioeconomic status (see main text for details).

Because there were small but significant differences in BMI values according to sex and SES, further analyses were required. Therefore, we assessed if there were significantly more women in an overweight/obese status within the three SESs. We found no significant differences in the proportion of men with or without abdominal obesity and excess weight (1.04 ratio) (χ_2__(1,3654)_ = 1.75, *p* = 0.186) among the SESs. In women, conversely, there was a higher number of individuals that met the criteria of obesity (ratio 1.96) (χ_2__(1,3654)_ = 433.75, *p* = 0.0001). When analyzing the proportion of women within each SES, the number of women with abdominal obesity and excess weight was significantly higher in the low (68%: χ_2__(1,2201)_ = 277.71, *p* = 0.0001), middle (64%: χ_2__(1,1521)_ = 123.756, *p* = 0.0001) and high SES (66%: χ_2__(1,379)_ = 36.64, *p* = 0.0001). In men, the overall ratios of participants with abdominal obesity and excess weight were barely above 1 (range: 0.92–1.32). Consequently, in the low SES, there were no differences (48%: χ_2__(1,2201)_ = 2.84, *p* = 0.092). However, for the middle (53%: χ_2__(1,1469)_ = 7.08, *p* = 0.008) and high SES (57%: χ_2__(1,357)_ = 6.98, *p* = 00.008), the differences were small yet significant (Figure 1).

## 4. Discussion

The current analyses of this LA multi-country representative urban survey indicate that people in the low SES show lower levels of diet quality (e.g., DDS, DQS and MAR), compared with the population with a more privileged socioeconomic status. Additionally, when analyzing anthropometric measurements by SES condition, we found a higher prevalence of abdominal obesity and excess weight in women.

Another relevant yet somewhat expected finding is that the consumption of fruits, vegetables, whole grains, fiber and fish and seafood increased progressively with income level. In addition, dairy foods were less consumed in the low SES, in comparison with the highest SESs. The only food group encouraged to be frequently consumed, which was higher in the low SES, was the legumes group.

In agreement with this evidence, our data showed that most healthy food groups are less consumed by individuals in the low SES. On the contrary, in LA countries, SSB beverages increase their consumption in the high SES. In the region, many sugar-containing beverages, both industrial and homemade, are highly consumed. The consumption of sugar-containing beverages has been previously published by our group, showing large between-countries differences with a typical pattern of high consumption [38].

Socioeconomically disadvantaged people have more difficulties to follow a healthy diet for many reasons, but one of the most obvious ones is its higher costs. Drenowsky and Darmon conducted a systematic review based on two hypotheses, the higher cost of a healthy diet and a poor nutritious diet of lower-income groups. They concluded that diets that are more nutritious tend to be more expensive and less affordable [39]. It is worth noting that diet costs could reflect either the intrinsic monetary cost of the diet or actual food expenditures [12].

Few studies have evaluated the differences in diet quality among socioeconomic subgroups in Latin American countries. A recent study that analyzed data from the Health Survey of São Paulo (ISA-Capital) comparing the Revised Brazilian Healthy Eating Index (BHEI-R) from 2003, 2008 and 2015, showed that lower-income individuals had a higher BHEI-R score in 2003. However, in 2008 and 2015, there was a shift in favor of higher-income individuals [40]. On the contrary, López-Olmedo et al. (2019) found that a lower educational level and lower assets index, used as indicators of SES, were positively associated with higher Mexican Diet Quality Index scores [41]. The authors reported that this observation might be explained by higher consumption of whole-grain cereals and legumes, characteristic of the traditional dietary pattern, among those in the lower SES. In agreement with the Mexican research, our study found higher consumption of legumes in the lower SESs, showing that it would be possible to improve the diet based on cultural and local food. Interesting, cultural differences, apparently, did not have a significative effect on diet quality across the Latin American region as socioeconomic status and gender did. Our results show the importance of taking into account sociodemographic factors in the design, interventions and food and health public policies.

It is expected that nutritious diets are associated with higher micronutrient adequacy. Our findings show that the higher the SES, the higher the MAR. We are aware that the differences are rather small among SESs; therefore, these data should be interpreted with caution. Nevertheless, the diet quality variables have been shown to be sensitive to different sociodemographic variables and are associated with nutritional and biometric parameters, suggesting that they are still informative in the context of nutritional studies [29], [34]. A positive association between income level and intake of calcium, vitamins C, E, D and MAR has been previously reported [42,43]. In line with our findings, the prevalence of inadequacy in the whole sample for vitamins D and E, with differences among SESs, has been observed in other populations [42,44]. Research has shown that adolescents and adults (≥15 years old) who are more affluent have relatively better intakes of vitamins and minerals, suggesting better overall diet quality [42,43]. There is a strong rationale to support the adverse social gradients in the population’s food choices and consumption, given the relation between core and discretionary foods and health, which may attenuate the disparity in food prices and diet costs [45].

Another study with the ELANS sample (9218) [46] has shown that participants were below the recommendations for most of the shortfall nutrients of public health concern analyzed (i.e., Vitamins A, D and E, calcium, potassium, folate, magnesium, iron and fiber). However, while not meeting the recommendations, most of the food sources, which were industrialized items, retain important nutrients of public health concern. Therefore, there is a dire need to strengthen the design, implementation and evaluation of strategies to increase access to healthy and industrialized food sources at an affordable cost for all income statuses.

It has been reported that low SES is associated with a higher risk of chronic diseases and mortality [47,48]. Furthermore, different approaches used to assess diet quality, e.g., the Healthy Eating Index (HEI), Alternate Healthy Eating Index and Dietary Approaches to Stop Hypertension Score, have reported that low diet quality is associated with a higher risk of all-cause mortality, cardiovascular disease, cancer, type 2 diabetes and neurodegenerative diseases [49]. Recently, the Global Burden of Diseases, Injuries and Risk Factor Study (GBD) (2017) assessed dietary data from geographically representative samples from 195 countries, highlighting the potential impact of a suboptimal diet on non-communicable diseases mortality and morbidity, in which the low consumption of whole grains, fruits and sodium accounted for more than 50% of deaths and 66% of disability-adjusted life-years attributable to diet [50].

Dietary quality indexes aim to evaluate the overall diet based on current nutrition knowledge and categorize individuals according to the extent to which their eating behavior is healthy [51]. In this study, we evaluated three different parameters of diet quality (e.g., DQS, DDS and MAR) and found that all of them were associated with a higher SES. These findings are consistent with previous studies conducted in other world regions. The US National Health and Nutrition Examination Survey reported higher adherence to the Dietary Guidelines for the Americans among adults with a higher income [52]. Similarly, the Japanese National Health and Nutrition Survey conducted in 2014 reported that low-income individuals with a low-quality diet were less likely to meet the Japanese dietary guidelines than higher-income individuals [53]. In most countries, diet quality was directly associated with SES. According to Damon and Drewnowski (2008), a lower diet quality index in the low-income population may be due to the limited resources to purchase healthier foods [54]. Evidence shows that food cost is one of the determinants of food choice, demonstrating the effect of a social gradient in diet quality [55]. Also, the neighborhood’s infrastructure could impact the access and availability of nutritionally dense foods; for example, Miller et al. (2016) found that the cost of fruits and vegetables is higher for people from low-income regions [56].

When analyzing the overall sample, our results show a direct association between dietary diversity and SES. These trends have also been identified in other population-based studies [57,58]. Dietary diversity ensures the consumption of vitamins, minerals and a wide range of bioactive substances, which have been linked with a reduced risk of inflammatory and malnutrition disorders [59].

Prevalence of overweight and obesity was higher among women and, among them, it was higher among those from the low-income group. These differences could be attributed to social determinants, such as gender and educational inequities. Education level is usually a proxy of economic status. In low- and middle-income levels, women tend to have lower education and even lower income, which affects some characteristics of the diet quality and diversity, leading to unhealthy dietary patterns. Those factors have been associated with higher prevalence of obesity in developing countries [40]. Wolongevics et al. (2010) concluded that inadequate diet quality predicted the development of overweight and obesity in women [60]. In addition, a study conducted with Brazilian women found a higher diet quality index and higher consumption of fruits, vegetables and whole grains in those with higher education levels. These authors suggest that higher-level education led women to adhere to a healthier diet [61].

The major strengths of this study include a large nationally representative sample of urban areas of eight LA countries. Two 24 h records assessed dietary intake and usual intake calculations enabled the minimization of the intra-individual variation in food consumption. Our study also has several limitations. First, as a cross-sectional study, the data should not be interpreted as the causal relationship between SES and diet quality. Second, the bias in food consumption measurements, including reliability in participants’ memory and the possible under- or overestimation of energy intake, should also be considered.

## 5. Conclusions

Our results showed a direct association between SES and diet quality, assessed by the diet quality score, the dietary diversity score and the nutrients adequacy ratio variables, showing that the higher the SES, the higher the scores of these variables. Participants in the low SES reported a lower consumption of dairy and nuts and seeds, while consumption of vegetables, fruits, whole grains and fiber, increased with SES. We also found a higher consumption of fish and seafood and a lower consumption of red meat and processed meat among those in the high SES. Women in the low SES showed a greater prevalence of abdominal obesity and excess weight than those in the middle and high SES. Social determinants of nutrition and health showed an association with dietary intake among Latin American individuals. Health policies and behavioral-change strategies should be addressed to reduce the impact of socioeconomic factors on compromising a healthy diet and nutritional status, with gender as an additional level of vulnerability.

## Figures and Tables

**Table 1:** Distribution of sociodemographic variables among the socioeconomic status—the Latin American Health and Nutrition Study (ELANS).

			Socioeconomic Levels						
	Total (*n* = 9218)		Low (*n* = 4796)		Middle (*n* = 3542)		High (*n* = 880)		
	*n*	%	*n*	%	*n*	%	*n*	%	*p* Value
Country									<0.001
Brazil	2000	21.7	916	19.1	915	25.8	169	25.8	
Venezuela	1132	12.3	880	18.3	190	5.4	62	5.4	
Colombia	1230	13.3	779	16.2	384	10.8	67	10.8	
Argentina	1266	13.7	616	12.8	585	16.5	65	16.5	
Peru	1113	12.1	533	11.1	355	10	225	10	
Chile	879	9.5	411	8.6	388	11	80	11	
Ecuador	800	8.7	399	8.3	297	8.4	104	8.4	
Costa Rica	798	8.7	262	5.5	428	12.1	108	12.1	
Sex									0.010
Male	4409	47.8	2221	46.3	1752	49.5	436	49.5	
Female	4809	52.2	2575	53.7	1709	50.5	444	50.5	
Age range (years)									0.441
15–19	1223	13.3	642	13.4	468	13.2	113	12.8	
20–34	3479	37.7	1803	37.6	1349	38.1	327	37.2	
35–49	2627	28.5	1332	27.8	1025	28.9	270	30.7	
50–65	1889	20.5	1019	21.2	700	19.8	170	19.3	

Percentages (%) correspond to the relative number of subjects per country within each socioeconomic status. *p* values correspond to chi-square (analysis). See main text for details.

**Table 2:** Consumption of food groups among the socioeconomic status—the Latin American Health and Nutrition Study (ELANS).

			Socioeconomic Status							
	Total (*n* = 9218)		Low (*n* = 4796)		Middle (*n* = 3542)		High (*n* = 880)			
	Mean	SD	Mean	SD	Mean	SD	Mean	SD	*p* Value	η^2^_p_
Vegetables (g)	105.6	54.1	100.3	51.4	109.4	56.2	119.8	56.2	0.0001	0.018
Fruits (g)	74.6	74.4	66.7	72.0	80.6	75.3	94.0	78.3	0.0001	0.013
Whole grains (g)	8.8	16.1	7.9	14.2	9.3	17.0	12.1	21.0	0.0001	0.007
Fiber (g)	15.8	5.8	15.6	5.8	15.6	5.9	16.9	5.7	0.0001	0.006
Legumes (g)	37.4	38.3	36.4	36.8	39.0	40.9	35.6	35.0	0.0001	0.004
Dairy (g)	94.2	94.6	89.3	90.2	100.0	99.7	97.6	95.6	0.0001	0.003
Red meat (g)	64.5	35.7	64.9	35.0	65.3	36.5	59.0	36.2	0.0001	0.003
Fish and seafood (g)	18.4	20.8	18.2	21.4	17.9	20.0	21.3	20.4	0.0001	0.002
SSB (g)	678.3	473.7	678.2	476.6	674.9	480.8	692.4	426.7	0.002	0.001
Processed meat (g)	19.5	16.4	19.1	16.3	20.3	16.5	17.7	16.0	0.005	0.001
Nuts and seeds (g)	2.1	9.0	1.9	9.8	2.1	8.6	2.5	5.7	0.049	0.001
Energy (Kcal)	1993.1	621.0	1987.7	631.6	1992.4	609.3	2025.3	608.6	0.255	0.000

Grams (g), standard deviation (SD), sugar-sweetened beverages (SSB). *p* values correspond to multivariate variance analyses comparing the three socioeconomic statuses, controlled by country, sex and age. See main text for details.

**Table 3:** Micronutrient adequacy ratio by socioeconomic status—the Latin American Health and Nutrition Study (ELANS).

			Socioeconomic Status							
	Total (*n* = 9218)		Low (*n* = 4796)		Middle (*n* = 3542)		High (*n* = 880)			
	Mean	SD	Mean	SD	Mean	SD	Mean	SD	*p* Value	η^2^_p_
Vitamin C	0.855	0.228	0.840	0.232	0.860	0.231	0.913	0.174	0.0001	0.010
Vitamin A	0.848	0.206	0.831	0.214	0.859	0.202	0.892	0.168	0.0001	0.009
Vitamin D	0.355	0.209	0.343	0.205	0.361	0.209	0.400	0.229	0.0001	0.007
Calcium	0.698	0.459	0.677	0.465	0.715	0.455	0.742	0.435	0.0001	0.003
Magnesium	0.764	0.184	0.759	0.185	0.765	0.183	0.792	0.176	0.0001	0.003
Copper	0.987	0.057	0.986	0.061	0.988	0.052	0.992	0.049	0.0001	0.001
Pyridoxin	0.972	0.087	0.970	0.087	0.972	0.087	0.980	0.073	0.0003	0.001
Iron	0.989	0.056	0.987	0.059	0.990	0.055	0.993	0.040	0.0005	0.001
Riboflavin	0.989	0.054	0.988	0.059	0.991	0.048	0.992	0.051	0.018	0.001
Thiamin	0.991	0.049	0.990	0.053	0.993	0.044	0.991	0.051	0.020	0.001
Cobalamin	0.985	0.076	0.983	0.081	0.987	0.071	0.988	0.067	0.041	0.001
Vitamin E	0.033	0.018	0.032	0.018	0.033	0.018	0.034	0.018	0.099	0.000
Selenium	0.999	0.021	0.999	0.024	1.000	0.010	0.999	0.034	0.214	0.000
Phosphorous	0.985	0.066	0.984	0.067	0.985	0.066	0.988	0.061	0.311	0.000
Zinc	0.965	0.091	0.964	0.091	0.966	0.091	0.968	0.089	0.437	0.000
Folate	0.656	0.183	0.654	0.184	0.657	0.181	0.658	0.182	0.677	0.000
Niacin	0.997	0.031	0.997	0.033	0.997	0.025	0.997	0.038	0.968	0.000
Mean adequacy ratio	0.828	0.063	0.823	0.065	0.831	0.062	0.842	0.057	0.001	0.010

Micronutrient adequacy ratio (mean consumption/estimated average requirements (EAR)). Standard deviation (SD). *p* values correspond to multivariate variance analyses comparing the three socioeconomic statuses, controlled by country, sex and age. See main text for details.

**Table 4:** Diet quality indicators by country according to socioeconomic status—the Latin American Health and Nutrition Study (ELANS).

			Socioeconomic Status							
	Total		Low		Middle		High			
	(*n* = 9218)		(*n* = 4796)		(*n* = 3542)		(*n* = 880)			
ELANS	Mean	SD	Mean	SD	Mean	SD	Mean	SD	*p* Value	η^2^_p_
Diet quality score	63.01	9.29	62.64	9.11	63.34	9.42	63.65	9.68	<0.001	0.002
Dietary diversity score	4.79	1.34	4.65	1.33	4.90	1.33	5.08	1.30	<0.001	0.012
Mean adequacy ratio	0.83	0.06	0.82	0.06	0.83	0.06	0.84	0.06	<0.001	0.007
**Argentina**	(*n* = 1266)		(*n* = 616)		(*n* = 585)		(*n* = 65)			
Diet quality score	63.47	9.56	62.84	9.09	63.85	9.95	66.03	10.01	<0.016	0.007
Dietary diversity score	4.48	1.30	4.33	1.28	4.62	1.33	4.72	1.19	<0.001	0.013
Mean adequacy ratio	0.83	0.04	0.82	0.04	0.83	0.04	0.85	0.03	<0.001	0.021
**Costa Rica**	(*n* = 798)		(*n* = 262)		(*n* = 428)		(*n* = 108)			
Diet quality score	63.46	9.41	62.97	9.51	63.72	9.30	63.58	9.66	>0.589	0.001
Dietary diversity score	4.97	1.35	4.70	1.27	5.08	1.37	5.20	1.35	<0.001	0.020
Mean adequacy ratio	0.80	0.06	0.79	0.06	0.81	0.06	0.83	0.05	<0.001	0.049
**Brazil**	(*n* = 2000)		(*n* = 916)		(*n* = 915)		(*n* = 169)			
Diet quality score	63.51	9.16	63.29	8.91	63.65	9.27	63.91	9.94	>0.596	0.001
Dietary diversity score	4.66	1.37	4.44	1.34	4.80	1.36	5.06	1.37	<0.001	0.023
Mean adequacy ratio	0.79	0.07	0.78	0.07	0.80	0.07	0.82	0.06	<0.001	0.026
**Chile**	(*n* = 879)		(*n* = 411)		(*n* = 388)		(*n* = 80)			
Diet quality score	61.42	10.33	59.5	10.34	62.81	10.18	64.18	9.35	<0.001	0.030
Dietary diversity score	4.78	1.20	4.75	1.26	4.78	1.16	4.95	0.99	>0.406	0.002
Mean adequacy ratio	0.79	0.07	0.79	0.07	0.80	0.06	0.81	0.06	<0.001	0.015
**Colombia**	(*n* = 1230)		(*n* = 779)		(*n* = 384)		(*n* = 67)			
Diet quality score	63.47	9.04	63.18	8.87	63.67	9.29	65.62	9.30	>0.093	0.004
Dietary diversity score	4.77	1.38	4.68	1.37	4.95	1.38	4.85	1.40	<0.007	0.008
Mean adequacy ratio	0.87	0.05	0.86	0.05	0.87	0.03	0.87	0.08	<0.008	0.008
**Peru**	(*n* = 1113)		(*n* = 533)		(*n* = 355)		(*n* = 225)			
Diet quality score	63.50	9.23	64.30	8.95	63.01	9.30	62.3	9.62	<0.015	0.007
Dietary diversity score	5.28	1.28	5.28	1.30	5.30	1.27	5.25	1.28	>0.880	0.000
MAR	0.85	0.05	0.85	0.05	0.85	0.05	0.86	0.05	<0.038	0.006
**Ecuador**	(*n* = 800)		(*n* = 399)		(*n* = 297)		(*n* = 104)			
Diet quality score	63.45	8.70	63.31	8.52	63.47	8.64	63.91	9.59	>0.820	0.000
Dietary diversity score	5.33	1.29	5.24	1.28	5.41	1.29	5.43	1.30	>0.153	0.005
Mean adequacy ratio	0.88	0.04	0.87	0.04	0.88	0.03	0.88	0.04	<0.009	0.012
**Venezuela**	(*n* = 1132)		(*n* = 880)		(*n* = 190)		(*n* = 62)			
Diet quality score	61.23	8.67	61.37	8.69	60.35	8.31	62.01	9.38	>0.259	0.002
Dietary diversity score	4.39	1.14	4.37	1.15	4.40	1.05	4.55	1.18	>0.484	0.001
Mean adequacy ratio	0.84	0.05	0.83	0.05	0.84	0.05	0.85	0.03	<0.009	0.008

Standard deviation (SD). *p* values correspond to multivariate variance analyses comparing the three socioeconomic statuses, controlled by sex and age. See main text for details.

**Table 5:** BMI among the socioeconomic statuses—the Latin American Health and Nutrition Study (ELANS).

	Total			Sex							
	ELANS			Men			Women				
Socioeconomic Status	Mean	SD	N	Mean	SD	N	Mean	SD	N	%	*p* Value
Low	26.92	5.71	879	26.01	5.11	2218	27.72	6.08	2572	6.17%	0.001
Middle	26.91	5.48	3539	26.52	5.22	1749	27.30	5.70	1790	2.87%	0.001
High	27.30	5.62	4790	27.10	5.62	436	27.49	5.62	443	1.40%	0.311
Total	26.96	5.62	9208	26.32	5.22	4403	27.54	5.90	4805	1.09%	0.0001

Standard deviation (SD). % indicates the proportion of women relative to men, expressed in percentages. *p* values correspond to multivariate variance analyses comparing the three socioeconomic levels by sex, controlled by country and age. See main text for details.

## Data Availability

Due to ethical and legal restrictions of the eight institutions involved, the data underlying this study are available upon request and must be approved by the Publishing Committee of ELANS. To apply for access to these data, interested researchers must submit a detailed project proposal. Requests for the data can be made to the correspondence author.
